# The Blind Spot of Small-Sided Games: Missing High-Intensity Deceleration Demands in Elite Soccer Players

**DOI:** 10.3390/sports14090370

**Published:** 2026-09-01

**Authors:** Vincenzo Manzi, Giuseppe Di Lascio, Manuele Taleb, Giuseppe Giardullo, Francesco Laterza, Cristian Savoia, Giuseppe Caminiti, Rosario D’Onofrio, Gaetano Raiola

**Affiliations:** 1 Department of Educational and Sport Sciences, Pegaso University of Naples, 80143 Naples, Italy; vincenzo.manzi@unipegaso.it (V.M.); francesco.laterza@univr.it (F.L.); donofrio.rosario@virgilio.it (R.D.); 2 Research Center of Physical Education and Exercise, University of Pegaso, 80143 Naples, Italy; giuseppe.dilascio@univr.it (G.D.L.); manuele.taleb@univr.it (M.T.); gaetano.raiola@unipegaso.it (G.R.); 3 Pisa Sporting Club, Serie A, 56124 Pisa, Italy; 4 Department of Neuroscience, Biomedicine and Movement, University of Verona, 37129 Verona, Italy; 5 The Research Institute for Sport and Exercise Sciences, The Tom Reilly Building, Liverpool John Moores University, Liverpool L3 5AH, UK; cristiansavoia@gmail.com; 6 Department of Human Science and Promotion of Quality of Life, San Raffaele Open University, 00166 Rome, Italy; giuseppe.caminiti@sanraffaele.it; 7 IRCCS San Raffaele, 00166 Rome, Italy

**Keywords:** small-sided games, high-intensity deceleration, accelerations, metabolic power, locomotor demands, external load, Jensen-Shannon divergence

## Abstract

Small-sided games (SSGs) are widely used in soccer training to replicate the technical, tactical, and physical demands of competition. However, their ability to reproduce the multidimensional distribution of accelerative and decelerative actions observed during official matches remains unclear. This study compared multidimensional locomotor distributions between SSGs and official match play in eleven professional soccer players monitored across more than 20 official matches and multiple SSG sessions during a competitive season. Locomotor data were obtained using validated tracking systems and analyzed by integrating speed, acceleration/deceleration magnitude, and metabolic power. The resulting multidimensional distributions were compared using Jensen–Shannon (JS) divergence to quantify distributional similarity. For accelerations, differences between SSGs and official matches were minimal at low metabolic power but increased progressively, reaching the greatest divergence above 60 W·kg^−1^ (JS = 0.196). Decelerations showed a similar trend; however, no deceleration events above 30 W·kg^−1^ were observed during SSGs, whereas official matches included exposure across higher metabolic power bands. These findings indicate that the SSG formats examined only partially reproduced the multidimensional locomotor demands of competition. Although accelerative actions remained represented across several metabolic power domains, exposure to high-intensity decelerations was absent during the SSG formats examined. From a practical perspective, these findings suggest that complementary training strategies may be considered when greater exposure to high-intensity deceleration demands is desired; however, the effectiveness of such strategies was not directly evaluated in the present study.

## 1. Introduction

Soccer is a complex intermittent sport characterized by unpredictable movement patterns and continuous transitions between low- and high-intensity activities. Elite players are required to repeatedly perform accelerations, decelerations, changes in direction, sprinting, and other high-intensity actions while maintaining technical and tactical performance throughout the match [1]. These movement demands impose substantial neuromuscular, metabolic, and mechanical stress, particularly during phases involving rapid accelerations and braking actions. Recent advances in performance analysis technologies, including high-frequency Global Positioning Systems (GPS), Local Positioning Systems (LPS), and semi-automated optical tracking systems, have enabled increasingly accurate quantification of external load variables such as running speed, acceleration/deceleration profiles, energy expenditure, and metabolic power during both training sessions and official match-play [2,3].

Over the last decade, small-sided games (SSGs) have become one of the most widely adopted training methodologies in elite soccer because they simultaneously integrate technical, tactical, physiological, and physical stimuli within representative game contexts [3]. Previous studies have shown that SSGs elicit high cardiovascular and metabolic responses, increase player involvement, and enhance technical decision-making [4,5]. However, growing evidence indicates that SSGs only partially reproduce the physical and locomotor demands experienced during official match-play [3,6]. Reduced pitch dimensions and constrained playing space appear to limit maximal running velocity and high-speed locomotor exposure while increasing the frequency of moderate accelerative and decelerative actions [3,7,8,9]. Consequently, metabolic power and kinematic profiles recorded during SSGs differ substantially from those observed during official competition, suggesting that apparently similar average locomotor values may conceal important differences in the organization of movement behaviours between training and match-play [6].

Among high-intensity locomotor actions, repeated decelerations have attracted increasing attention because they are associated with elevated eccentric loading, neuromuscular fatigue, muscle damage, and a potentially greater mechanical load than accelerations of comparable magnitude [10]. Nevertheless, most previous investigations comparing SSGs with official match-play have focused primarily on isolated speed thresholds, acceleration counts, or average locomotor metrics, without considering the multidimensional interaction between speed, acceleration/deceleration magnitude, and metabolic power. Recent evidence has suggested that analyses based exclusively on speed categories provide only a partial representation of the actual locomotor and physiological demands encountered during soccer activities [11]. Similarly, acceleration intensity should be interpreted in relation to the corresponding running speed, since acceleration capacity progressively decreases as velocity increases [12]. These findings indicate that locomotor demands cannot be fully characterized using isolated variables alone. Metabolic power models provide an integrated framework for quantifying locomotor intensity because they simultaneously account for running speed and acceleration/deceleration demands during intermittent exercise [2,13]. However, relying exclusively on average metabolic power or traditional locomotor variables may still mask substantial differences in the multidimensional organization of movement behaviours between training and competition [6]. Consequently, similar average values between SSGs and official match-play do not necessarily indicate similar locomotor demands.

To overcome this limitation, probabilistic approaches derived from information theory have recently been proposed to compare entire locomotor distributions rather than isolated variables or average values. Unlike conventional analyses, these approaches compare complete probability distributions, thereby preserving information on how locomotor events are organized across multiple intensity domains. Accordingly, Jensen–Shannon (JS) divergence provides a robust information-theoretic framework for quantifying similarities and differences between multidimensional movement distributions [14]. This approach enables the simultaneous evaluation of locomotor behaviour across metabolic power, running speed, and acceleration/deceleration magnitude, thereby providing a more comprehensive characterization of external load than traditional unidimensional analyses.

Therefore, the aim of the present study was to compare the multidimensional distributions of accelerative and decelerative actions between official match-play and SSGs in elite professional soccer players using a Jensen–Shannon divergence approach. We hypothesized that SSGs would only partially reproduce the multidimensional locomotor demands of official match-play, with progressively greater divergence expected as metabolic power increased and the largest discrepancies anticipated for high-intensity decelerative actions.

## 2. Materials and Methods

### 2.1. Experimental Approach to the Problem

This study was designed as an observational comparative analysis aimed at investigating whether small-sided games (SSGs) reproduce the multidimensional locomotor demands observed during official matchplay in professional soccer players. In particular, the study focused on the distribution of accelerations and decelerations across different metabolic power, speed, and acceleration/deceleration domains. Players were monitored during seven months of full training (including pre-season and in-season) and over 20 matches (Elite official matches). All players underwent the same type of training sessions 7 times a week throughout the preseason, with a friendly match played on a Thursday or during the weekend. Training sessions were mainly devoted to technical-tactical skill development, with fitness training sessions performed as a single training modality (i.e., no other exercises) during pre-season. The training time during the observed period was devoted to ball drills (28%) and generic aerobic training (5%); 32% and 14% of the training time was spent on technical-tactical skill development and matches, respectively. Explosive power training was performed 2 times a week as a single morning session (i.e., plyometric training, 5%). The remaining time was spent with warm-up routines (16%). The SSGs were performed using different pitch dimensions (24 × 16 m to 40 × 32 m) and player formats ranging from 4v4 to 6v6, similarly to previous investigations examining the physiological and kinematic demands of SSGs in elite soccer players [3,4]. A detailed description of the SSG formats, pitch dimensions, training prescriptions, distribution, and playing rules is provided in Section 2.3 and Table 1.

During championship weeks, players trained 6 times per week with one Elite match usually occurring on a Sunday or Saturday. Only players who regularly participated in both official matches and SSG sessions were included in the analysis.

Goalkeepers’ tracking data were excluded from the analysis due to their specific locomotor demands. Accelerative and decelerative actions were analyzed separately in order to identify possible action-specific discrepancies between training and competition demands.

### 2.2. Participants

Eleven (25.7 ± 2.9 years, height 183.1 ± 4.1 cm and body mass 79.5 ± 6.8 kg) elite male professional Italian Elite soccer players (2 central defenders, 2 wide defenders, 2 central midfielders, 2 box-to-box midfielders, 2 wingers and 1 striker volunteered to take part in this study. All players had at least seven years of experience playing in national championships from junior age. Each professional player gave informed consent regarding the use of results observed during their usual training sessions and official matches for research purposes. All participants were informed about the study protocol and gave their informed consent to participate. This study was approved by the ethical principles set forth by the Centre’s Ethics Committee, under protocol code PROT/E 004726 dated 15 July 2025. All the procedures involved in this study were in accordance with the Declaration of Helsinki.

### 2.3. Small-Sided Games Protocol

A total of 40 SSG training sessions were analyzed during the seven-month observation period. Because more than one SSG format could be implemented within the same training session, 53 format-specific SSG occurrences were identified. A format-specific occurrence was defined as the implementation of a given SSG format for a player group within a training session. Each occurrence comprised three consecutive bouts.

Of the 53 occurrences, 20 involved the 4v4 format, 15 the 5v5 format, and 18 the 6v6 format, corresponding to 37.7%, 28.3%, and 34.0% of all occurrences, respectively. The resulting occurrence-based ratio between the 4v4, 5v5, and 6v6 formats was 20:15:18. As each occurrence comprised three bouts, a total of 159 SSG bouts were analyzed: 60 in the 4v4 format, 45 in the 5v5 format, and 54 in the 6v6 format.

Across the 11 outfield players included in the study, 227 valid player-format exposures were recorded. A player-format exposure was counted when a player participated in a specific SSG format during a training session; repeated bouts within the same format-specific occurrence were not counted as separate exposures. Of the 227 exposures, 107 occurred during 4v4 games, 54 during 5v5 games, and 66 during 6v6 games, corresponding to 47.1%, 23.8%, and 29.1% of all player-format exposures, respectively. Because not all players participated in every SSG occurrence, individual exposure varied across players and formats.

The 4v4 games were played on 24 × 16 m or 30 × 20 m pitches, corresponding to relative playing areas of 48 and 75 m^2^ per outfield player, respectively. The 5v5 games were played on 28 × 20 m or 35 × 25 m pitches, corresponding to 56 and 88 m^2^ per outfield player, respectively. The 6v6 games were played on 32 × 24 m or 40 × 32 m pitches, corresponding to 64 and 107 m^2^ per outfield player, respectively. Relative playing area was calculated according to the total number of outfield players and therefore did not include goalkeepers.

Bout duration ranged from 2 to 4 min for the 4v4 format and from 3 to 5 min for the 5v5 and 6v6 formats. Consecutive bouts were separated by 1–3 min of passive recovery.

Goalkeepers participated only in the 5v5 and 6v6 formats, whereas the 4v4 games were performed without goalkeepers. Goalkeepers’ tracking data were excluded from the locomotor analysis. Depending on the specific objective of the training session, the SSGs were performed with one-, two-, or three-touch limitations or under free-play conditions. No jokers or neutral players were used. The 4v4 format was played using small goals, whereas the 5v5 and 6v6 formats involved goalkeepers and regulation-sized goals. The offside rule was applied in the 5v5 and 6v6 formats but not in the 4v4 games. When the ball left the playing area, or following a goal, play was restarted according to standard football rules. Constant verbal encouragement was provided by the coaching staff throughout all SSG bouts. Format-specific characteristics, training prescriptions, and player-format exposures are summarized in Table 1.

### 2.4. Data Collection and Locomotor Analysis

Locomotor activity during training sessions and small-sided games was monitored using K-AI wearable tracking devices (K-Sport World S.R.L., Pesaro, Italy), equipped with a 50 Hz differential GNSS receiver (GPS, GLONASS, Galileo, and BeiDou) and an integrated inertial measurement unit comprising tri-axial accelerometer, gyroscope, and magnetometer sensors. Official match locomotor data were collected using the Hawk-Eye optical tracking system (Hawk-Eye Innovations Limited, Basingstoke, UK).

Although training and match data were acquired using different tracking technologies, all positional data were subsequently processed within the Dynamix analytical platform (K-Sport World S.R.L.), which is designed to acquire, standardize, and analyse tracking inputs originating from different tracking technologies through a common analytical framework [15,16].

Tracking data from both sources were imported, standardized, and analyzed using Dynamix software (version 1.25.6; K-Sport World S.R.L., Pesaro, Italy), which has previously been adopted for the processing and analysis of elite football tracking data [15] and has recently shown excellent agreement between real-time and post-processed external-load metrics derived from league optical tracking data [16].

Accordingly, the present study compared standardized locomotor variables generated within a common analytical framework rather than directly comparing proprietary outputs from the two acquisition technologies.

The validity and reliability of high-frequency GNSS systems for assessing locomotor demands and sprint-related mechanical variables in team sports have previously been reported [17,18].

External load data were classified according to metabolic power categories based on the model proposed by di Prampero et al. [13] and subsequently adapted to soccer by Osgnach et al. [2]. Metabolic power categories provide an integrated estimation of the energetic cost associated with the interaction between speed and acceleration/deceleration during intermittent locomotor activities [2,19]. The metabolic power thresholds adopted in the present study were selected a priori according to the classification proposed by Osgnach et al. [2], which represents one of the most widely adopted frameworks for describing locomotor intensity in elite soccer using the metabolic power approach. Their use was intended to ensure methodological consistency and facilitate direct comparison with previous investigations employing the same classification. Metabolic power zones were selected to provide a progressive characterization of locomotor intensity across different energetic domains, consistently with previous metabolic power investigations in soccer. Metabolic power bands were categorized as 0–10, 10–20, 20–30, 30–40, 40–50, 50–60, and >60 W·kg^−1^. Within each metabolic power band, locomotor actions were further classified according to speed (0–8, 8–13, 13–16, 16–19, 19–22, 22–25, and >25 km·h^−1^) and acceleration/deceleration magnitude (0 to −0.5, −0.5 to −1, −1 to −2, −2 to −3, −3 to −4, −4 to −5, and <−5 m·s^−2^). This multidimensional approach was adopted to provide a more comprehensive characterization of locomotor demands than traditional analyses based exclusively on isolated speed thresholds or average values [10,20]. For each condition (official matches and SSGs), normalized multidimensional distributions were computed to describe the relative occurrence of accelerative and decelerative events within the interaction of metabolic power, speed, and acceleration/deceleration domains. Accelerations and decelerations were analyzed separately.

### 2.5. Distribution Analysis

To compare locomotor distributions between official matches and small-sided games (SSGs), Jensen–Shannon (JS) divergence was used as a probabilistic measure of distribution dissimilarity. JS divergence is a symmetric and bounded measure derived from Shannon entropy that quantifies differences between multidimensional probability distributions [14]. Unlike conventional analyses based on average values or isolated thresholds, JS divergence compares the entire probability distribution of locomotor observations, thereby preserving information on how movement demands are organized across multiple intensity domains. Consequently, distribution-based methods provide a more comprehensive characterization of locomotor behaviour than traditional mean-based analyses, particularly when complex interactions between speed, acceleration/deceleration magnitude, and metabolic power are considered.

For each metabolic power band, separate two-dimensional histograms were constructed for official matches and SSGs, with speed categories represented on one axis and acceleration or deceleration magnitude categories on the other. Each histogram therefore consisted of a 7 × 7 matrix (49 cells). Although the overall locomotor framework comprised three dimensions (metabolic power, speed, and acceleration/deceleration), JS divergence was calculated separately within each metabolic power band using the corresponding two-dimensional speed-by-acceleration (or speed-by-deceleration) matrix. The predefined bin boundaries corresponded to the categories described in Section 2.3, and no data-dependent binning procedure was applied. The use of predefined thresholds ensured that the discretization scheme was established independently of the observed data, thereby avoiding data-driven optimization of the probability distributions and preserving direct comparability with previous studies using the metabolic power framework. The raw tracking data consisted of consecutive time samples acquired at a fixed sampling interval. Each time sample contained synchronized measurements of running speed, metabolic power, and acceleration/deceleration. The multidimensional analysis was therefore performed at the time-sample level rather than by identifying discrete acceleration or deceleration events. Each time sample was independently classified according to the predefined metabolic power, speed, and acceleration/deceleration categories.

For each experimental condition (official matches and SSGs), all classified time samples from all eligible players and all corresponding observations were pooled to construct a single multidimensional frequency matrix within each metabolic power band. No weighting procedures based on playing time, number of classified time samples, or individual player contribution were applied. Consequently, each classified time sample contributed equally to the empirical distribution, irrespective of the player from whom it originated.

For each condition, the resulting frequency matrix was flattened into a 49-element vector and normalized by dividing each cell count by the total number of classified time samples within the corresponding metabolic power band. This procedure generated two discrete probability distributions, P (official matches) and Q (SSGs), each summing to one. The midpoint probability distribution was calculated as:

$$JSD\left(P,Q\right)=\frac{1}{2}\sum_{i=1}^{49}p_{i}ln\;\left(\frac{p_{i}}{m_{i}}\right)+\frac{1}{2}\sum_{i=1}^{49}q_{i}ln\;\left(\frac{q_{i}}{m_{i}}\right),\;M=\frac{P+Q}{2}.$$

Zero-frequency cells were retained in the distributions. Terms associated with zero probabilities were assigned a contribution of zero according to the convention $0ln\left(0/m\right)=0$. No pseudocounts, epsilon corrections, kernel density estimation, or other smoothing procedures were applied. When an entire metabolic power band contained no SSG observations, the corresponding probability distribution could not be normalized, and JS divergence was therefore considered not estimable for that band.

The computational procedure was implemented in Python using NumPy for matrix handling and normalization and the scipy.spatial.distance.jensenshannon function from the SciPy library for distribution comparison. Because this function returns the Jensen–Shannon distance (i.e., the square root of the JS divergence), its output was squared to obtain the JS divergence reported in the present study:

$$JSD\left(P,Q\right)={\left[jensenshannon\left(P,Q,base=None\right)\right]}^{2}$$

The computational procedure was applied independently to accelerative and decelerative distributions within each metabolic power band. Heat-map visualizations were generated from the normalized 7 × 7 matrices to illustrate the relative occurrence of locomotor events across the speed and acceleration/deceleration domains. Similar probabilistic approaches have recently been proposed in sport performance analysis to investigate movement-pattern organization and external-load distributions [21].

To quantify the sampling uncertainty associated with the JS divergence estimates, a hierarchical non-parametric bootstrap procedure with 10,000 iterations was subsequently performed. During each iteration, players were sampled with replacement and, within each selected player, official matches and SSG sessions were independently resampled with replacement. For each bootstrap sample, multidimensional distributions were reconstructed following the same computational procedure described above, normalized to probability distributions, and JS divergence was recalculated. Bootstrap standard errors (SE) were estimated as the standard deviation of the bootstrap distribution, whereas percentile-based 95% confidence intervals (95% CI) were obtained from the 2.5th and 97.5th percentiles of the bootstrap distribution. Confidence intervals were reported only for estimable JS values.

Lower JS values indicated greater similarity between official match-play and SSG locomotor distributions, whereas higher values reflected greater divergence between the two conditions. The analysis focused on identifying:

1. Whether SSGs reproduced the multidimensional structure of match locomotor demands;
2. Which metabolic power domains exhibited the greatest discrepancies;
3. Whether accelerative and decelerative actions showed different distributional behaviors between training and competition.

### 2.6. Statistical Analysis

Descriptive statistics and multidimensional locomotor distribution summaries were generated from datasets collected during official matches and SSGs. Jensen–Shannon (JS) divergence analyses were performed in Python (version 3.14.6; Python Software Foundation, Wilmington, DE, USA). Python using the SciPy library, following the computational procedure described in Section 2.5. Sampling uncertainty was quantified using the hierarchical non-parametric bootstrap procedure described above, with bootstrap standard errors (SE) and percentile-based 95% confidence intervals (95% CI) reported for all estimable JS divergence values. When no SSG observations were recorded within an entire metabolic power band, JS divergence and the corresponding uncertainty estimates were considered not estimable. JS divergence values were interpreted descriptively, with progressively larger values indicating greater divergence between official match-play and SSG locomotor distributions.

## 3. Results

**Figure 1:**
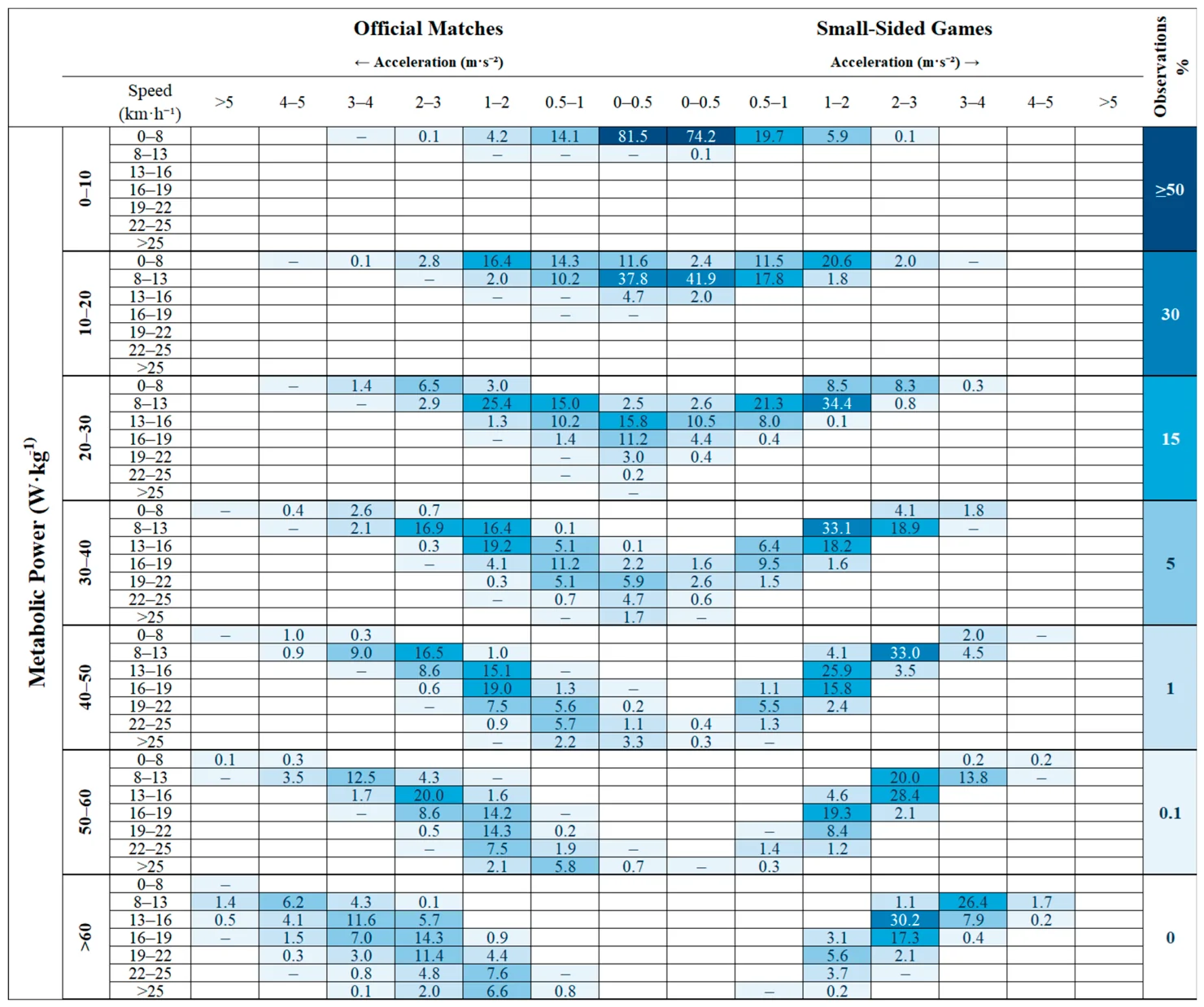
Distribution of observations across speed, acceleration, and metabolic power zones during official matches (OM; left side, acceleration magnitude increases leftward from the center) and small-sided games (SSGs; right side, acceleration magnitude increases rightward from the center). Values are expressed as percentages of classified time samples within each metabolic power band. Colors represent predefined discrete percentage intervals. Darker blue categories indicate a greater proportion of classified time samples. The symbol “–” indicates values > 0 but <0.1%, whereas blank cells indicate the absence of observations (0%).

**Figure 2:**
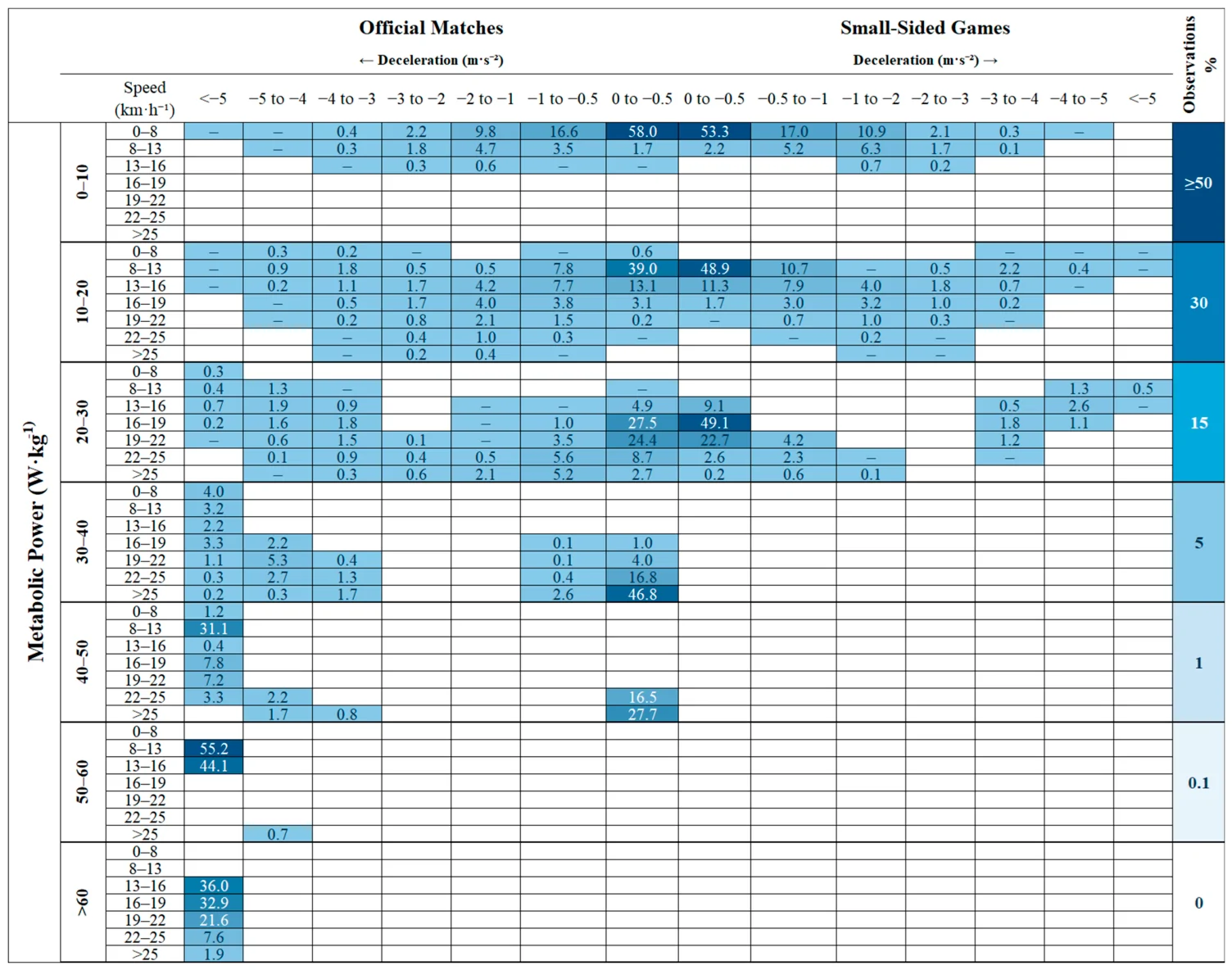
Distribution of observations across speed, deceleration magnitude, and metabolic power zones during official matches (OM; deceleration magnitude increases leftward from the center) and small-sided games (SSGs; deceleration magnitude increases rightward from the center). Values are expressed as percentages of classified time samples within each metabolic power band. Colors represent predefined discrete percentage intervals. Darker blue categories indicate a greater proportion of classified time samples. The symbol “–” indicates values > 0 but <0.1%, whereas blank cells indicate the absence of observations (0%). Deceleration categories are displayed using their original signed intervals for consistency with the predefined classification scheme.

**Figure 3:**
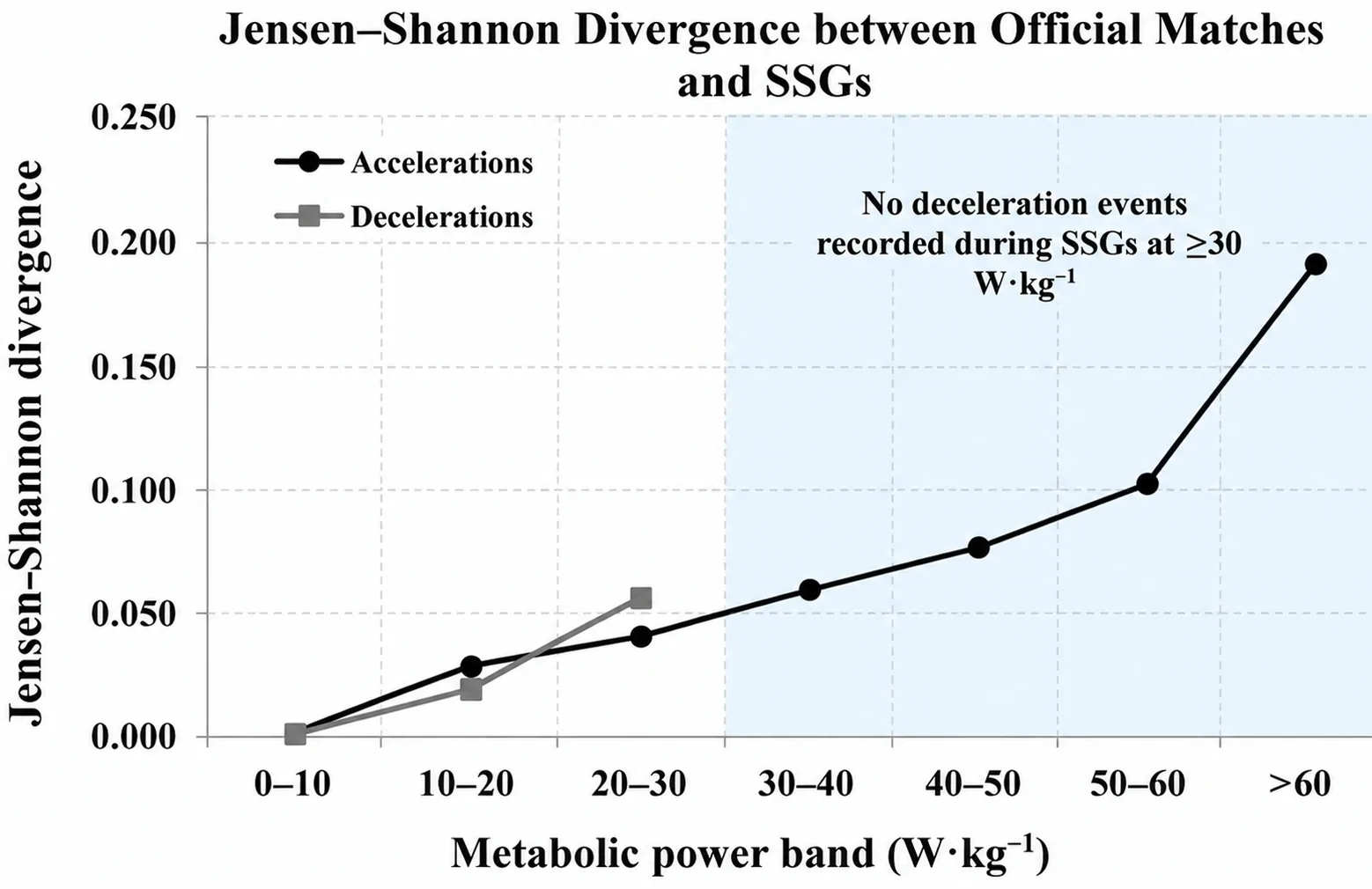
Jensen–Shannon (JS) divergence between multidimensional locomotor distributions observed during official match-play and small-sided games across metabolic power bands. Lower JS values indicate greater similarity between official match-play and SSG locomotor distributions. No deceleration events were recorded during SSGs at metabolic power intensities ≥ 30 W·kg^−1^; consequently, JS divergence could not be estimated beyond this threshold. Bootstrap standard errors (SE) and percentile-based 95% confidence intervals (95% CI) for all estimable JS values are reported in Table 2 and Table 3.

The multidimensional distribution analysis illustrated by the heat maps (Figure 1 and Figure 2), together with the Jensen–Shannon (JS) divergence analysis (Figure 3) and the corresponding bootstrap uncertainty estimates (Table 2 and Table 3), revealed clear differences between official matches and SSGs, with distinct distributional behaviours for accelerative and decelerative actions across metabolic power bands.

### 3.1. Accelerative Actions

For accelerative actions, the lowest metabolic power band showed minimal divergence between official matches and SSGs (0–10 W·kg^−1^; JS = 0.004, 95% CI: 0.002–0.007), indicating a highly similar distribution of low-intensity accelerative events. JS divergence increased progressively with metabolic power, reaching 0.029 (95% CI: 0.025–0.035) in the 10–20 W·kg^−1^ band and 0.040 (95% CI: 0.032–0.050) in the 20–30 W·kg^−1^ band. At higher metabolic power intensities, divergence increased further (0.059, 95% CI: 0.049–0.072; 0.076, 95% CI: 0.063–0.097; and 0.102, 95% CI: 0.087–0.131 for the 30–40, 40–50, and 50–60 W·kg^−1^ bands, respectively), reaching the highest value in the >60 W·kg^−1^ band (JS = 0.196, 95% CI: 0.161–0.252). These findings indicate that although accelerative actions were represented across all metabolic power domains during SSGs, their multidimensional distribution became progressively less similar to that observed during official match-play as locomotor intensity increased. The corresponding multidimensional distributions are illustrated in Figure 1, whereas the bootstrap uncertainty estimates are reported in Table 2.

### 3.2. Decelerative Actions

For decelerative actions, JS divergence was minimal in the lowest metabolic power band (0–10 W·kg^−1^; JS = 0.003, 95% CI: 0.002–0.006), indicating a similar distribution of low-intensity braking actions between official matches and SSGs. Divergence increased to 0.020 (95% CI: 0.013–0.033) in the 10–20 W·kg^−1^ band and reached 0.056 (95% CI: 0.043–0.089) in the 20–30 W·kg^−1^ band, indicating progressively greater differences in the multidimensional distribution of moderate-intensity decelerative actions. No deceleration events were recorded during SSGs at metabolic power intensities ≥ 30 W·kg^−1^. Consequently, the corresponding probability distributions could not be constructed, and JS divergence was not estimable for these bands. The multidimensional distribution patterns are shown in Figure 2, whereas the corresponding bootstrap uncertainty estimates are summarized in Table 3.

Figure 3 summarizes the JS divergence observed across metabolic power bands for accelerative and decelerative actions. For accelerations, JS divergence increased progressively with metabolic power, demonstrating that SSGs became progressively less representative of official match-play as locomotor intensity increased. Bootstrap-derived confidence intervals confirmed the robustness of this progressive increase across all estimable metabolic power bands. In contrast, decelerative actions exhibited only modest divergence up to 20–30 W·kg^−1^, after which no SSG events were recorded, precluding further distributional comparison. This absence of high-intensity deceleration exposure represents the principal distributional mismatch between SSGs and official match-play observed in the present study.

## 4. Discussion

The main finding of the present study was that SSGs only partially reproduced the multidimensional locomotor structure of official matchplay in elite soccer players. While accelerative actions were still represented across several metabolic power domains, high-intensity deceleration demands were markedly underrepresented or completely absent during SSGs, particularly above 30 W·kg^−1^. These findings suggest that the main discrepancy between SSGs and official matches lies in the inability of SSGs to reproduce peak brake demands under high metabolic power conditions. Previous investigations comparing SSGs and official matchplay have already suggested that training games may fail to fully reproduce competition demands, especially regarding high-intensity locomotor activities [3,22]. Reduced pitch dimensions and constrained spatial environments have been shown to decrease high-speed running exposure while increasing the frequency of short accelerative and decelerative actions. Similarly, Dalen et al. [23] reported that smaller-sided formats increased moderate acceleration and deceleration frequency but failed to reproduce the highest running intensities observed during official matches. However, most previous studies relied on isolated speed thresholds, acceleration counts, or average locomotor metrics. The present investigation extends previous evidence by adopting a multidimensional framework integrating speed, acceleration/deceleration magnitude, and metabolic power simultaneously. The relative preservation of accelerative actions during SSGs may be explained by the constrained spatial characteristics of these drills, which promote frequent changes in direction and repeated accelerative efforts. Small-sided formats require players to constantly reinitiate movement over short distances, resulting in a high density of moderate accelerative actions. Because braking intensity is strongly dependent on entry velocity and momentum dissipation, the spatial constraints imposed by reduced playing areas may inherently limit exposure to the most demanding decelerative actions. As a result, players may be unable to reach the elevated running velocities typically required to generate high-intensity braking loads. This may explain why deceleration exposure progressively disappeared in SSGs above 30 W·kg^−1^ despite accelerative actions still being represented within higher metabolic power domains. These findings are particularly relevant considering the mechanical demands associated with intense decelerations. Based on previous literature, high-intensity decelerations have been associated with increased eccentric loading and elevated neuromuscular demands. However, the present study quantified locomotor distributions using a metabolic power approach and did not directly assess mechanical braking forces, joint loading, or muscle damage. Therefore, these physiological implications should be interpreted as evidence derived from previous research rather than findings demonstrated by the present investigation [24]. However, these physiological mechanisms were not directly assessed in the present investigation and therefore should be interpreted as evidence derived from previous research rather than findings of the current study. Recent evidence has also shown that deceleration exposure may impose substantial neuromuscular and musculoskeletal stress, potentially contributing to fatigue accumulation and injury risk [25]. Therefore, the absence of high metabolic power deceleration events during SSGs may represent an important training limitation, potentially leading to insufficient exposure to the peak braking loads encountered during official competition. Another important aspect emerging from the present study concerns the methodological approach used to characterize locomotor demands. Recent studies have highlighted that analyses based exclusively on speed categories may provide only a partial representation of the actual physiological and locomotor demands encountered during soccer activities [26]. Similarly, average metabolic power values may conceal substantial differences in the organization and distribution of movement behaviors between training and competition scenarios [27]. The present findings support this perspective by demonstrating that apparently similar locomotor intensities may emerge from profoundly different acceleration/deceleration structures. The robustness of these distributional patterns was further supported by the bootstrap-derived uncertainty estimates, which confirmed the progressive increase in Jensen–Shannon divergence across metabolic power bands for accelerative actions while reinforcing the absence of estimable high-intensity deceleration distributions during SSGs. In particular, the multidimensional distribution analysis revealed that the discrepancy between SSGs and official matches became progressively larger as metabolic power increased, especially for decelerative actions. The present findings also align with recent methodological approaches emphasizing the importance of contextualizing accelerative actions according to their locomotor characteristics rather than relying exclusively on isolated acceleration thresholds. Sonderegger et al. [12] demonstrated that acceleration intensity should be interpreted in relation to initial running speed because acceleration capacity decreases as running speed increases. In this context, the multidimensional approach adopted in the present study may provide a more ecologically valid representation of locomotor demands by simultaneously integrating speed, acceleration/deceleration magnitude, and metabolic power within the same analytical framework. From a practical perspective, coaches and practitioners should be aware that SSGs alone may not provide sufficient exposure to the highest intensity deceleration demands experienced during official matches. Consequently, coaches may consider complementing SSG-based training with drills specifically designed to increase exposure to high-speed deceleration scenarios. However, the effectiveness of such training strategies was not directly evaluated in the present investigation and therefore should be regarded as a practical implication rather than an experimentally demonstrated conclusion. While SSGs remain an effective training strategy for developing technical, tactical, and moderate-intensity locomotor actions, the present findings suggest a potential ‘blind spot’ in the specific SSG formats examined regarding the reproduction of high-intensity braking exposure observed during official match-play. Overall, our results indicate that multidimensional distribution analysis based on Jensen–Shannon divergence represents a robust methodological framework for evaluating the representativeness of small-sided games relative to official match-play while identifying specific locomotor demands that may remain insufficiently reproduced during training. This approach may assist researchers and practitioners in designing training tasks that more closely replicate the multidimensional locomotor demands of competitive match-play. Some limitations of the present study should be acknowledged. First, the analysis was conducted on a relatively small sample of elite professional players from a specific competitive context, which may limit the generalizability of the findings. Second, official matches and SSGs were monitored using different tracking technologies (optical tracking and GNSS, respectively). Although all locomotor variables were processed within the same analytical framework, subtle differences related to data acquisition and processing cannot be completely excluded and may have contributed, to some extent, to the observed differences. Third, different SSG formats were grouped together, and the potential influence of pitch size, player number, and tactical constraints was not analyzed separately. In addition, analyses were performed on the overall sample without positional stratification. Although locomotor demands are known to differ among playing positions, the limited number of players within each positional subgroup did not allow robust distributional comparisons. Fourth, although the metabolic power approach provides an integrated description of locomotor intensity, its application across movement contexts characterized by different locomotor patterns (i.e., official matches and SSGs) should be interpreted with caution. Furthermore, the predefined metabolic power thresholds were selected a priori according to the framework proposed by Osgnach et al. to ensure methodological consistency and comparability with previous studies. Although alternative discretization schemes could produce different absolute Jensen–Shannon divergence values, evaluating the sensitivity of the results to alternative threshold definitions was beyond the scope of the present investigation. Fifth, contextual match variables, including opponent quality, scoreline, ball possession, accumulated fatigue, and environmental conditions, were not specifically controlled. Because the present study was designed to compare overall multidimensional locomotor distributions across an extended monitoring period rather than to isolate the independent contribution of contextual factors, these variables may have contributed to part of the observed variability. Finally, only external locomotor demands were considered, without integrating internal load, neuromuscular markers, or biomechanical measures of braking load.

Future studies should investigate whether specific SSG configurations or complementary training drills can better reproduce the high-intensity deceleration demands observed during official competition. In addition, future research involving larger cohorts should evaluate positional differences in multidimensional locomotor distributions, examine the influence of contextual match variables, and determine whether alternative metabolic power discretization schemes produce comparable distributional patterns.

## 5. Conclusions

In conclusion, small-sided games only partially reproduced the multidimensional locomotor demands of official soccer matches. While accelerative actions were relatively preserved across several metabolic power domains, exposure to high-intensity deceleration demands was absent during SSGs at metabolic power intensities ≥ 30 W·kg^−1^. These findings highlight a potential “blind spot” of SSG-based training and reinforce the need for complementary training drills to reproduce the peak braking demands encountered during official competition. Furthermore, the present study demonstrates that multidimensional distribution analysis based on Jensen–Shannon divergence provides a robust methodological framework for quantifying the representativeness of SSGs relative to official match-play and for identifying specific locomotor demands that may remain insufficiently reproduced during training. These findings should be interpreted within the specific context of the player sample, SSG formats, tracking technologies, and analytical procedures adopted in the present study.

## Figures and Tables

**Table 1:** Format-specific SSG configuration and training prescription.

SSG Format	Small Pitch (m)	Medium Pitch (m)	Area Per Player (m^2^; Small/Medium)	Total Analyzed Bouts	Bout Duration, Range (Mean)	Passive Recovery (min)
4v4	24 × 16	30 × 20	48/75	20	2–4 (3 min)	1–3
5v5	28 × 20	35 × 25	56/88	15	3–5 (4 min)	1–3
6v6	32 × 24	40 × 32	64/107	18	3–5 (4 min)	1–3

**Table 2:** Bootstrap uncertainty estimates for Jensen–Shannon (JS) divergence across metabolic power bands during accelerative actions. JS divergence values quantify the multidimensional differences between locomotor distributions observed during official matches and small-sided games. Bootstrap standard errors (SE) and percentile-based 95% confidence intervals (95% CI) were estimated from 10,000 hierarchical non-parametric bootstrap iterations performed on the athlete-level dataset.

Metabolic Power (W·kg^−1^)	JS Divergence	Bootstrap SE	95% CI
0–10	0.004	0.001	0.002–0.007
10–20	0.029	0.003	0.025–0.035
20–30	0.040	0.005	0.032–0.050
30–40	0.059	0.006	0.049–0.072
40–50	0.076	0.009	0.063–0.097
50–60	0.102	0.011	0.087–0.131
>60	0.196	0.023	0.161–0.252

**Table 3:** Bootstrap uncertainty estimates for Jensen–Shannon (JS) divergence across metabolic power bands during decelerative actions. JS divergence values quantify the multidimensional differences between locomotor distributions observed during official matches and small-sided games. Bootstrap standard errors (SE) and percentile-based 95% confidence intervals (95% CI) were estimated from 10,000 hierarchical non-parametric bootstrap iterations performed on the athlete-level dataset. Confidence intervals are reported only for estimable JS values. JS divergence was not estimable for metabolic power bands ≥ 30 W·kg^−1^ because no deceleration events were recorded during SSGs, preventing construction of a normalized probability distribution.

Metabolic Power (W·kg^−1^)	JS Divergence	Bootstrap SE	95% CI
0–10	0.003	0.001	0.002–0.006
10–20	0.020	0.005	0.013–0.033
20–30	0.056	0.012	0.043–0.089
30–40	Not estimable	—	—
40–50	Not estimable	—	—
50–60	Not estimable	—	—
>60	Not estimable	—	—

## Data Availability

Data can be obtained from the corresponding author upon reasonable request.
